# Screen-detected raised blood pressure and gaps in the hypertension care cascade among adults attending a humanitarian medical mission in Ukraine: A cross-sectional study

**DOI:** 10.1371/journal.pone.0357957

**Published:** 2026-09-11

**Authors:** Kuan-Ting Chen, En-Tse Yen, Kun-Zhe Tsai, Shih-Ying Lin, Regina Maistrova, Weide Tsai

**Affiliations:** 1 Division of Cardiology, Department of Internal Medicine, MacKay Memorial Hospital, Taipei, Taiwan; 2 International Medical Service Center, MacKay Memorial Hospital, Taipei, Taiwan; 3 Department of Orthodontics, MacKay Memorial Hospital, Taipei, Taiwan; 4 Graduate School of Oral Medicine, Matsumoto Dental University, Shiojiri, Japan; 5 Department of Stomatology, MacKay Memorial Hospital, Taipei, Taiwan; 6 MacKay Medical University, New Taipei City, Taiwan; 7 Department of Periodontology, School of Dentistry, Tri-Service General Hospital and National Defense Medical Center, Taipei, Taiwan; 8 Department of Dentistry, MacKay Memorial Hospital, Taipei, Taiwan; 9 School of Dentistry, College of Medicine, National Taiwan University, Taipei, Taiwan; 10 Christian Medical Association of Ukraine, Lviv, Ukraine; National University, SUDAN

## Abstract

Cardiovascular disease is the world’s leading cause of death, and raised blood pressure is among its most important modifiable contributors. Conflict tends to interrupt the long-term care that hypertension demands, yet little screening data exist from settings of active conflict. We set out to describe screen-detected raised blood pressure, and the hypertension care cascade, among adults seen at a humanitarian medical mission in Ukraine. In this cross-sectional study we included adults (≥ 18 years) seen consecutively at a MacKay Memorial Hospital mission across three Ukrainian sites in April 2026. Blood pressure was taken once, and we counted a participant as having raised blood pressure if that single reading was ≥ 140/90 mmHg or they reported a previous hypertension diagnosis. Prevalence is reported with Wilson 95% confidence intervals and standardized to the World Health Organization world population; adjusted prevalence ratios come from modified Poisson regression with robust variance. For the cardiovascular screening stream we also described awareness, treatment, and control. Of 364 adults (mean age 52.5 years; 63.2% women), 65.7% of the 341 with complete outcome data had raised blood pressure on the crude estimate (95% confidence interval 60.5–70.5), falling to 54.7% (48.1–61.3) after age standardization. Prevalence climbed with age and was higher in men. Older age (adjusted prevalence ratio 1.19 per 10 years, 1.11–1.27), male sex (1.26, 1.08–1.47), and obesity (1.24, 1.09–1.42) were each independently associated with the outcome. Within the screening stream, 74.6% of those with raised blood pressure were aware, 58.8% were treated, and just 15.8% were controlled; estimates held across multiple imputation and alternative specifications. This was a self-selected sample assessed on a single reading and cannot speak to population prevalence, but the pattern—common raised blood pressure with poor control—makes a reasonable case for building simple screening and a clear referral pathway into missions of this kind.

## Introduction

Cardiovascular disease remains the leading cause of death worldwide, and raised blood pressure is one of its most important modifiable contributors [1]. The number of adults living with hypertension has roughly doubled over the past three decades. Many remain un-diagnosed; many who are diagnosed are untreated or treated without reaching control; and these gaps fall hardest on low- and middle-income settings [2]. The WHO has made hypertension a priority for global action and has set out practical steps for managing it in primary care [3,4].

Conflict and displacement strain health systems, and chronic noncommunicable diseases, which depend on continuity rather than acute intervention, are among the first to suffer [5]. Drug supplies are interrupted, records are lost, and acute needs crowd out routine follow-up; at the same time, stress, dietary change, and reduced activity can push cardiovascular risk higher [5,6]. Among populations affected by conflict or displacement, studies have repeatedly found a heavy burden of hypertension alongside clear shortfalls in treatment [7,8].

The war in Ukraine has, since 2022, kept up sustained pressure on the country’s health services and on the people living in affected areas. One way that residents and displaced people reach care is through short-term humanitarian missions, and those visits double as a chance to pick up cardiovascular risk that has gone undetected or under-treated. Yet blood-pressure screening data from missions in active conflict are scarce, and we know little about how many attendees are aware of their hypertension, are being treated, or have it under control.

Against that background, we analyzed cardiovascular screening data from adults seen at a MacKay Memorial Hospital humanitarian mission in Ukraine in April 2026. We set out to estimate how common screen-detected raised blood pressure was, to work out which factors were associated with it, and to describe awareness, treatment, and control among attendees of the mission’s cardiovascular screening stream. Because the design is a single screening encounter, we read our results as raised blood pressure on one occasion, not as confirmed hypertension.

## Materials and methods

### Study design and setting

This was a cross-sectional analysis of screening data from a short-term humanitarian mission that MacKay Memorial Hospital (Taipei, Taiwan) ran with local partners in Ukraine in April 2026. The mission worked at three sites, Korosten, Zvyagel, and Ternopil, and ran several parallel streams: a cardiovascular screening and referral stream (which we refer to as “TRAP”), a traditional Chinese medicine (TCM) stream, and a dental stream. We report the study in line with the Strengthening the Reporting of Observational Studies in Epidemiology (STROBE) recommendations for cross-sectional studies [9] (S1 Checklist).

### Ethics

MacKay Memorial Hospital’s Institutional Review Board approved the study (approval number 25MMHIS075e; approved 19 May 2025), which we conducted according to the Declaration of Helsinki. Every participant gave written informed consent, together with the study questionnaire, before taking part.

### Inclusivity in global research

Additional information regarding the ethical, cultural, and scientific considerations specific to inclusivity in global research is included in the Supporting Information (S2 Checklist).

### Participants

Anyone aged 18 or older who attended the mission and had their blood pressure measured was eligible. People came of their own accord, so the sample is a self-selected convenience sample rather than a population-based one. We had records for 372 individuals; after removing 8 who were under 18, 364 adults remained for analysis.

### Measurements

Trained mission staff took a single seated blood-pressure reading from each participant, along with heart rate. They measured height and weight to calculate body-mass index (BMI; kg/m^2^) and classified obesity as BMI ≥ 30 kg/m^2^. Age, sex, smoking status (never, former, or current), and alcohol use came from interview. Participants seen in the cardiovascular stream answered additional questions: about any prior hypertension diagnosis, current use of blood-pressure-lowering medication, diabetes, established cardiovascular disease, family history, and usual sleep duration.

### Definitions

Our primary outcome was raised blood pressure, which we defined as a single screening reading of systolic ≥ 140 mmHg or diastolic ≥ 90 mmHg, or a self-reported prior diagnosis of hypertension, a composite definition [10]. Since blood pressure was taken only once, this captures raised blood pressure at one encounter and is not the same as a clinical diagnosis of hypertension, which needs repeated readings on separate occasions. For a sensitivity analysis we also used a measured-only definition (≥ 140/90 mmHg at the visit, regardless of any reported diagnosis). Among cardiovascular-stream participants with raised blood pressure, we defined awareness as a self-reported prior diagnosis, treatment as current use of blood-pressure-lowering medication, and control as a measured blood pressure below 140/90 mmHg.

### Statistical analysis

We summarized continuous variables as means (with standard deviations, SD) and categorical variables as counts and percentages, comparing sites and streams with analysis of variance or the χ² test as appropriate. Prevalence estimates carry Wilson 95% CIs [11]. To make the figures easier to compare with other populations, we also standardized prevalence to the WHO world standard population by direct standardization [12]. For the associations with raised blood pressure we used modified Poisson regression with robust (sandwich) variance, which suits binary outcomes that are common and sidesteps the inflation that follows from reading odds ratios as risk ratios [13,14]. The main model held age (per 10 years), sex, obesity, current smoking, alcohol use, site, and stream. A second model, limited to cardiovascular-stream participants with raised blood pressure, looked at what was associated with being on treatment. We described the cascade, awareness, treatment, and control, in that same group, again with Wilson CIs.

Several sensitivity analyses were specified in advance: modeling BMI continuously (per 5 kg/m^2^) rather than as an obesity threshold; switching to the measured-only outcome; restricting the data to the cardiovascular stream; and handling missing values with multiple imputation by chained equations (20 imputations), combined under Rubin’s rules [15]. As a further check we refit the main model with log-binomial regression. The blood-pressure outcome was missing for about 6% of adults. We treated a two-sided P below 0.05 as significant and ran the analyses in Stata 19 (StataCorp, College Station, TX, USA) [16].

## Results

### Participants

The 364 adults averaged 52.5 years of age (SD 14.1), and 230 (63.2%) were women (Table 1). Mean BMI was 28.6 kg/m^2^ (5.6), and 133 (36.5%) met the obesity threshold. Just under one in ten (9.9%) were current smokers and 23.1% reported drinking alcohol. The Ternopil attendees stood out as younger, with more men and more current smokers than at Korosten or Zvyagel (Table 1). Across the three streams, 147 (40.4%), 134 (36.8%), and 83 (22.8%) adults were enrolled, differing modestly in age and alcohol use (S1 Table).

**Table 1:** Characteristics of adult mission attendees, by recruitment site.

Characteristic	Korosten	Zvyagel	Ternopil	Total	P
No. of participants	106 (29.1)	94 (25.8)	164 (45.1)	364 (100.0)	
Age, years, mean (SD)	56.6 (14.0)	54.6 (13.3)	48.7 (13.7)	52.5 (14.1)	<0.001
*Sex, n (%)*					<0.001
Women	77 (72.6)	69 (73.4)	84 (51.2)	230 (63.2)	
Men	29 (27.4)	25 (26.6)	80 (48.8)	134 (36.8)	
BMI, kg/m², mean (SD)	28.6 (5.7)	28.9 (5.2)	28.3 (5.8)	28.6 (5.6)	0.670
Obesity (BMI ≥ 30), n (%)	37 (34.9)	37 (39.4)	59 (36.0)	133 (36.5)	0.792
Systolic BP, mmHg, mean (SD)	146.0 (23.3)	139.3 (20.4)	134.8 (22.8)	139.2 (22.8)	<0.001
Diastolic BP, mmHg, mean (SD)	86.4 (11.7)	87.5 (10.0)	88.7 (11.8)	87.7 (11.4)	0.296
Heart rate, bpm, mean (SD)	80.4 (15.5)	78.7 (16.0)	80.1 (12.0)	79.8 (14.0)	0.707
*Smoking status, n (%)*					<0.001
Never	96 (90.6)	86 (91.5)	118 (72.0)	300 (82.4)	
Former	8 (7.5)	5 (5.3)	15 (9.1)	28 (7.7)	
Current	2 (1.9)	3 (3.2)	31 (18.9)	36 (9.9)	
Alcohol use, n (%)	11 (10.4)	17 (18.1)	56 (34.1)	84 (23.1)	<0.001
Raised blood pressure, n (%)	68 (66.7)	59 (72.8)	97 (61.4)	224 (65.7)	0.204

Data are mean (SD) or n (%). BP, blood pressure; BMI, body-mass index; SD, standard deviation. Raised blood pressure was defined as a single screening reading ≥140/90 mmHg or a self-reported prior diagnosis of hypertension. Percentages for raised blood pressure are among participants with complete outcome data (n = 341). P values are from analysis of variance for continuous variables and the χ^2^ test for categorical variables. Blood pressure was measured once at a single visit. MacKay Memorial Hospital humanitarian mission, Ukraine, April 2026.

### Prevalence of raised blood pressure

**Fig 1:**
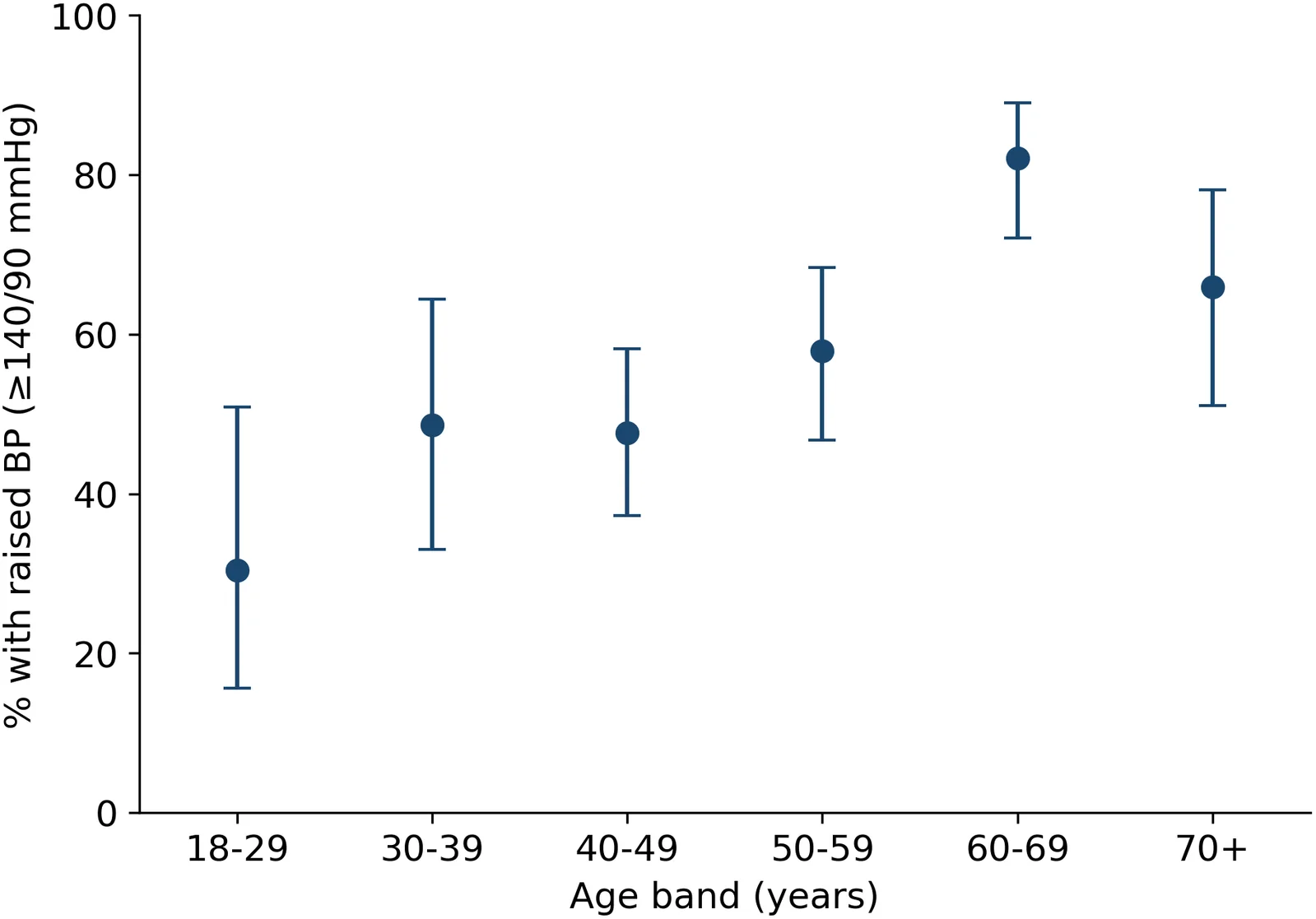
Age-specific prevalence of raised blood pressure. Points are the prevalence of measured raised blood pressure (≥ 140/90 mmHg) by 10-year age band and vertical lines are Wilson 95% confidence intervals. Based on a single office reading. MacKay Memorial Hospital humanitarian mission, Ukraine, April 2026.

Crude prevalence of screen-detected raised blood pressure was 65.7% (95% CI 60.5–70.5), among the 341 adults with complete outcome data. Using the measured threshold alone (≥ 140/90 mmHg) it was 59.1% (53.8–64.2). Standardizing to the WHO world population pulled these down to 54.7% (48.1–61.3) and 50.1% (43.5–56.8), respectively, which reflects how much older the mission sample was. Using the measured-only definition, prevalence climbed across most age bands, from 30.4% (15.6–50.9) at 18–29 years to 82.1% (72.1–89.0) at 60–69 years (Fig 1). Measured raised blood pressure was a little more common in the cardiovascular stream (62.3%) than in the TCM (59.7%) or dental (50.0%) streams.

### Factors associated with raised blood pressure

**Fig 2:**
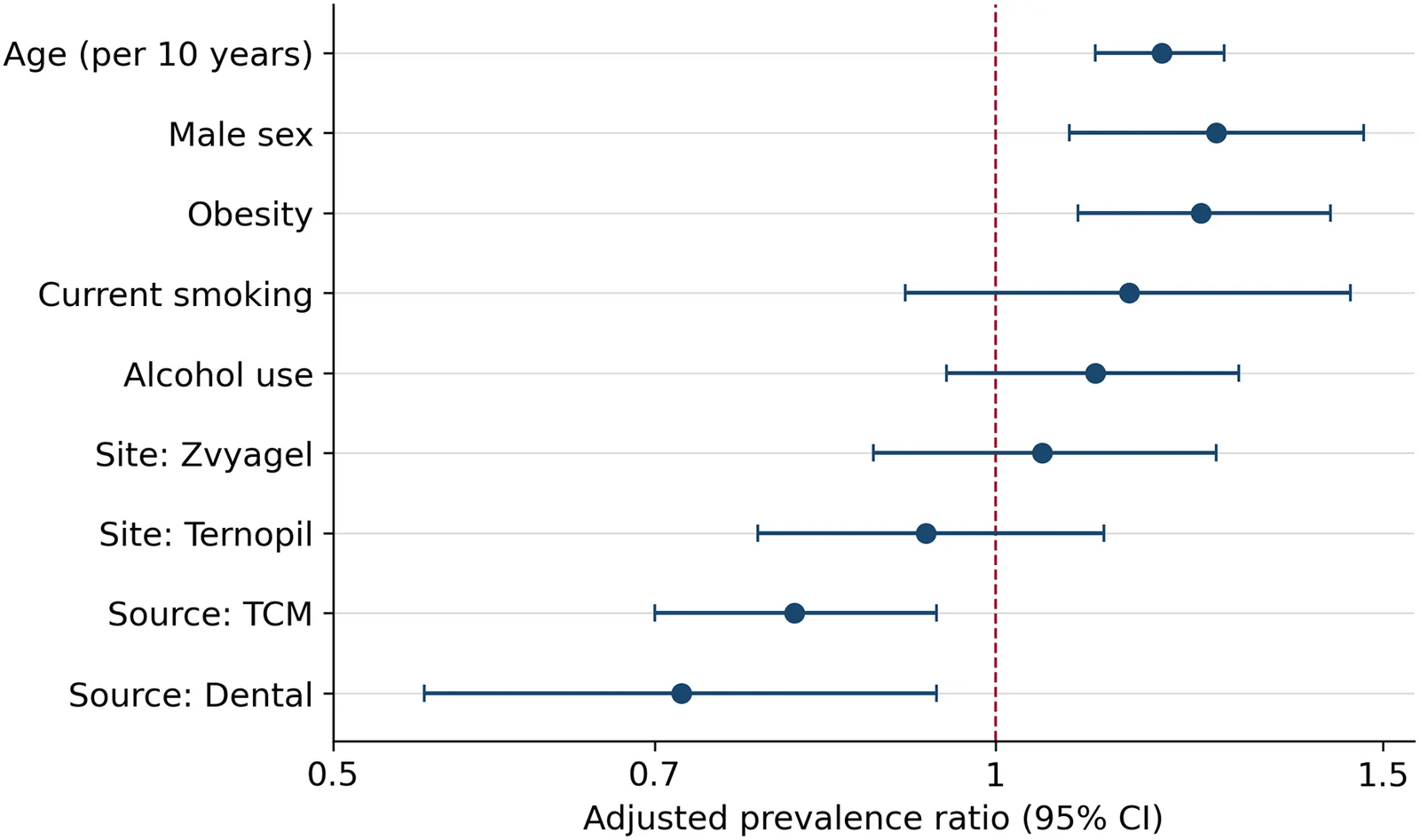
Adjusted prevalence ratios for raised blood pressure. Estimates are from a modified Poisson regression model with robust variance among all adults (n = 341). Points are adjusted prevalence ratios and horizontal lines are 95% confidence intervals; the dashed vertical line marks the null value of 1. Reference categories are female sex, non-obese, never/former smoking, no alcohol use, Korosten (site), and the cardiovascular-focused stream (source). The horizontal axis is on a logarithmic scale.

After adjustment, prevalence rose with age (aPR 1.19 per 10 years, 95% CI 1.11–1.27) and was higher in men (1.26, 1.08–1.47) and in obese participants (1.24, 1.09–1.42) (Table 2, Fig 2). Current smoking (1.15, 0.91–1.45) and alcohol use (1.11, 0.95–1.29) showed no clear association. Site made no significant difference once other factors were accounted for. Participants in the TCM (0.81, 0.70–0.94) and dental (0.72, 0.55–0.94) streams had a lower prevalence of the composite outcome than the cardiovascular stream, a gap that, as shown below, narrowed once we used the measured-only definition.

**Table 2:** Adjusted prevalence ratios for raised blood pressure and for current treatment.

Variable	Raised BP, aPR (95% CI)	On treatment, aPR (95% CI)
Age (per 10 years)	1.19 (1.11–1.27)***	1.10 (0.95–1.27)
Male sex	1.26 (1.08–1.47)**	0.96 (0.69–1.33)
Obesity	1.24 (1.09–1.42)**	1.25 (0.91–1.70)
Current smoking	1.15 (0.91–1.45)	—
Alcohol use	1.11 (0.95–1.29)	—
Site: Zvyagel	1.05 (0.88–1.26)	—
Site: Ternopil	0.93 (0.78–1.12)	—
Source: TCM	0.81 (0.70–0.94)**	—
Source: dental	0.72 (0.55–0.94)*	—
**No. of observations**	**341**	**114**

aPR, adjusted prevalence ratio; BP, blood pressure; CI, confidence interval; TCM, traditional Chinese medicine. Estimates are from modified Poisson regression with robust variance. The raised-blood-pressure model includes all adults; the treatment model is restricted to participants with raised blood pressure in the cardiovascular-focused stream. Reference categories are female sex, non-obese, never/former smoking, no alcohol use, Korosten (site), and the cardiovascular-focused stream (source). An em dash (—) indicates a covariate not included in the treatment model. * P < 0.05; ** P < 0.01; *** P < 0.001.

### Care cascade

**Fig 3:**
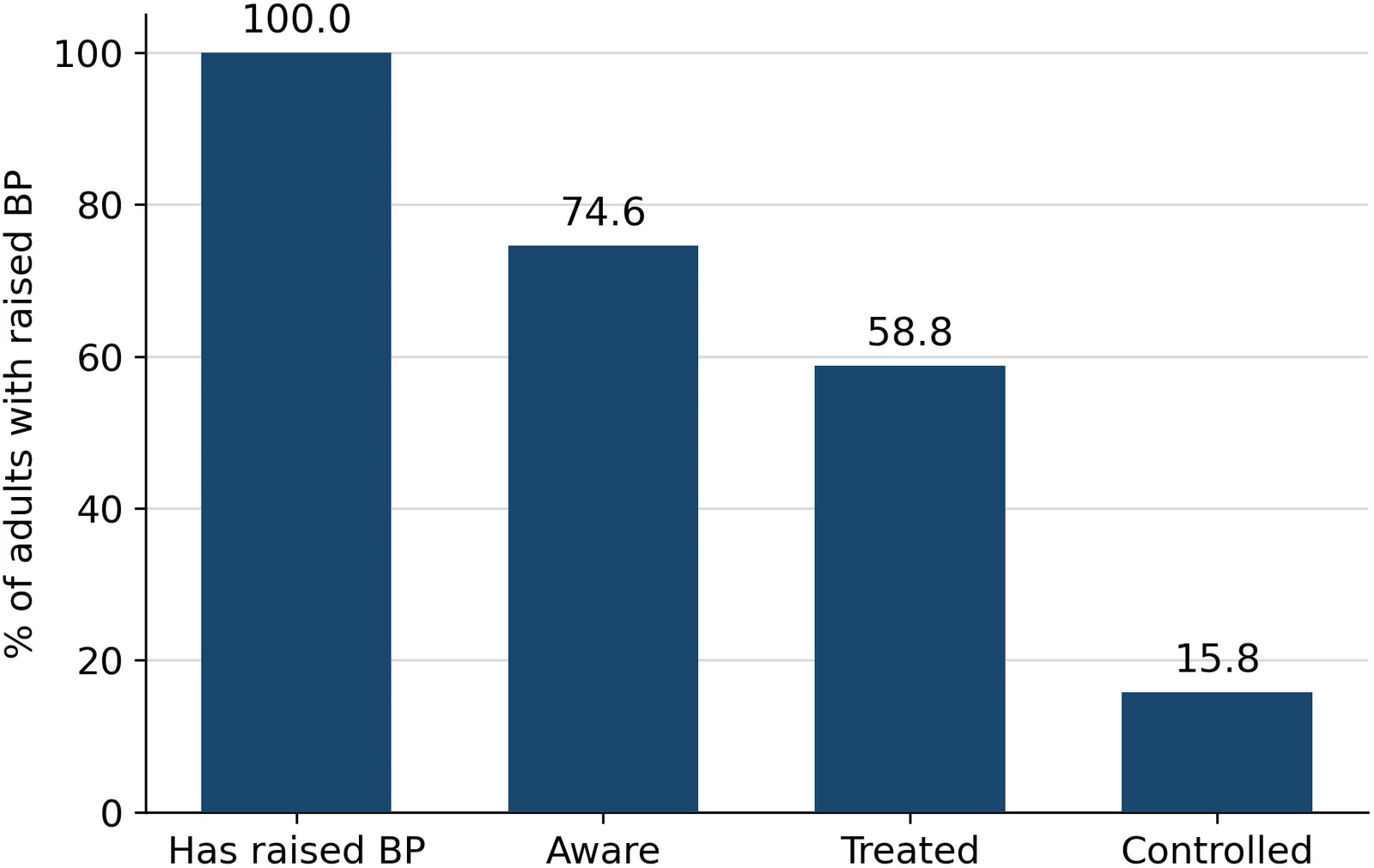
Blood-pressure care cascade among screening-stream adults with raised blood pressure. Bars show the percentage of participants with raised blood pressure in the cardiovascular-focused stream (n = 114) who were aware of a prior diagnosis, currently treated with blood-pressure-lowering medication, and controlled (measured blood pressure below 140/90 mmHg). Based on a single office reading. MacKay Memorial Hospital humanitarian mission, Ukraine, April 2026.

Of the 114 cardiovascular-stream participants with raised blood pressure, 74.6% (95% CI 65.9–81.7) were aware of a prior diagnosis, 58.8% (49.6–67.4) were taking blood-pressure-lowering medication, and only 15.8% (10.2–23.6) had a measured blood pressure under 140/90 mmHg (Fig 3). Among those aware of their diagnosis, 70.6% (60.2–79.2) were treated; among those treated, 26.9% (17.7–38.5) were controlled. The biggest losses came at treatment and control: 43.0% of everyone with raised blood pressure was treated but still above target, and 84.2% was uncontrolled overall. Control looked like it might differ by site, but the numbers in each cell were small and the difference fell short of significance (Fisher’s exact P = 0.069), so we treat it as hypothesis-generating only.

In the treatment model, restricted to cardiovascular-stream participants with raised blood pressure, neither age, sex, nor obesity was significantly tied to being on treatment (Table 2).

### Sensitivity analyses

The age, sex, and obesity associations barely moved when BMI was modeled continuously (1.12 per 5 kg/m^2^, 1.05–1.19) or under multiple imputation (S2 Table). With the measured-only definition, those three associations held, but the lower prevalence in the TCM and dental streams faded to nothing (TCM 0.99, 0.83–1.18; dental 0.90, 0.68–1.20). The most likely reading is that the stream differences in the composite outcome came mostly from who reported a prior diagnosis, not from differences in measured blood pressure. A log-binomial model pointed the same way.

## Discussion

Among adults attending a humanitarian mission in Ukraine, screen-detected raised blood pressure was common: roughly two-thirds on the crude estimate, and about half once we standardized for age. It tracked with older age, male sex, and obesity. Within the cardiovascular stream, awareness was reasonably high, but treatment and, above all, control lagged well behind.

That burden sits comfortably alongside what others have reported in conflict-affected and displaced populations [5,7,8], and alongside the global picture in which a large fraction of people with hypertension are not controlled [1,2]. The shape of the cascade, awareness largely intact but heavy losses at treatment and control, is what one would expect when medication access and routine follow-up are disrupted, as they are in humanitarian settings [5,6].

Where control is this low, the practical message for mission planning is fairly direct. Awareness was already high, so the gains are to be found less in detection and more in starting or stepping up treatment and in supporting adherence and follow-up. A brief mission may get more value from simple, standardized screening tied to an explicit handover to local care, an approach in keeping with WHO guidance for cardiovascular care in primary settings [3,4], than from a one-off measurement.

The way the stream differences behaved is itself telling. Because they disappeared under the measured-only definition, the gap across streams in the composite outcome came mainly from differential reporting of a prior diagnosis, presumably reflecting who chose to attend cardiovascular services, rather than from any real difference in measured pressure. It is a reminder to keep measured and self-reported components apart when reading screening data.

This analysis has some real strengths: two outcome definitions (composite and measured-only); prevalence ratios from modified Poisson regression rather than odds ratios [13,14]; age standardization for external comparison [12]; and a set of sensitivity analyses, multiple imputation among them, that left the estimates largely intact [15]. Contemporaneous cardiovascular screening data from missions in active conflict are relatively sparse, and ours may be useful for planning future missions.

The limitations matter, and several are important. Above all, blood pressure was measured only once; the outcome is therefore screen-detected raised blood pressure, and it will overstate the prevalence of confirmed hypertension, which depends on repeated readings. The sample selected itself, these were people who chose to attend, so it does not represent the wider population of the regions served, and the prevalence figures should not be read that way. The cascade analysis sat within the cardiovascular stream and rested on small numbers, which limited precision and ruled out firm site comparisons. Awareness, treatment, smoking, and alcohol use were all self-reported, with the recall and social-desirability biases that brings. Outcome data were missing for about 6% of adults, probably not at random; multiple imputation gave consistent results, but some residual bias may remain. With only three sites, we treated site as fixed, so any site-level reading is exploratory. And being cross-sectional, the study cannot establish that any of these associations is causal.

## Conclusions

Among adults at a humanitarian mission in Ukraine, screen-detected raised blood pressure was common and control was poor, with the widest gaps at the treatment and control ends of the cascade. These data come from a self-selected sample and a single reading, and they do not represent the general population, but they do make a reasonable case for building simple, standardized hypertension screening, and a clear link to ongoing local care, into humanitarian missions in conflict-affected settings.

## Supporting information

S1 Table Characteristics of adult mission attendees, by service stream. (DOCX)

S2 Table Sensitivity analyses for factors associated with raised blood pressure. (DOCX)

S1 Checklist STROBE checklist for cross-sectional studies. (DOCX)

S2 Checklist PLOS questionnaire on inclusivity in global research. (DOCX)
