# Supplementary material for: Screen-detected raised blood pressure and gaps in the hypertension care cascade among adults attending a humanitarian medical mission in Ukraine: A cross-sectional study
Source: PLoS One. 2026 Sep 11;21(9):e0357957. doi: 10.1371/journal.pone.0357957 (PMC13567746; doi:10.1371/journal.pone.0357957)
Supplement: Supplementary file 1 [file pone.0357957.s001.docx]

**S1 Table. Characteristics of adult mission attendees, by service stream.**

| **Characteristic** | **TRAP** | **TCM** | **Dental** | **Total** | **P** |
| --- | --- | --- | --- | --- | --- |
| No. of participants | 147 (40.4) | 134 (36.8) | 83 (22.8) | 364 (100.0) |  |
| Age, years, mean (SD) | 54.5 (13.9) | 53.0 (14.1) | 48.3 (13.6) | 52.5 (14.1) | 0.004 |
| *Sex, n (%)* |  |  |  |  | 0.515 |
| Women | 95 (64.6) | 87 (64.9) | 48 (57.8) | 230 (63.2) |  |
| Men | 52 (35.4) | 47 (35.1) | 35 (42.2) | 134 (36.8) |  |
| BMI, kg/m², mean (SD) | 29.0 (5.6) | 28.1 (5.2) | 28.6 (6.2) | 28.6 (5.6) | 0.385 |
| Obesity (BMI ≥30), n (%) | 59 (40.1) | 41 (30.6) | 33 (39.8) | 133 (36.5) | 0.199 |
| Systolic BP, mmHg, mean (SD) | 141.3 (22.8) | 139.0 (24.0) | 134.6 (19.5) | 139.2 (22.8) | 0.166 |
| Diastolic BP, mmHg, mean (SD) | 88.6 (11.0) | 87.0 (12.3) | 87.1 (10.1) | 87.7 (11.4) | 0.484 |
| Heart rate, bpm, mean (SD) | 80.3 (15.5) | 78.9 (12.3) | 80.9 (14.1) | 79.8 (14.0) | 0.597 |
| *Smoking status, n (%)* |  |  |  |  | 0.491 |
| Never | 124 (84.4) | 108 (80.6) | 68 (81.9) | 300 (82.4) |  |
| Former | 7 (4.8) | 14 (10.4) | 7 (8.4) | 28 (7.7) |  |
| Current | 16 (10.9) | 12 (9.0) | 8 (9.6) | 36 (9.9) |  |
| Alcohol use, n (%) | 43 (29.3) | 33 (24.6) | 8 (9.6) | 84 (23.1) | 0.003 |
| Raised blood pressure, n (%) | 114 (77.6) | 80 (59.7) | 30 (50.0) | 224 (65.7) | <0.001 |

Data are mean (SD) or n (%). BP, blood pressure; BMI, body-mass index; SD, standard deviation; TCM, traditional Chinese medicine; TRAP, cardiovascular-focused screening and referral stream. Raised blood pressure was defined as a single screening reading ≥140/90 mmHg or a self-reported prior diagnosis of hypertension. P values are from analysis of variance for continuous variables and the χ² test for categorical variables. Blood pressure was measured once at a single visit. MacKay Memorial Hospital humanitarian mission, Ukraine, April 2026.
