# Supplementary material for: Screen-detected raised blood pressure and gaps in the hypertension care cascade among adults attending a humanitarian medical mission in Ukraine: A cross-sectional study
Source: PLoS One. 2026 Sep 11;21(9):e0357957. doi: 10.1371/journal.pone.0357957 (PMC13567746; doi:10.1371/journal.pone.0357957)
Supplement: Supplementary file 2 [file pone.0357957.s002.docx]

**S2 Table. Sensitivity analyses for factors associated with raised blood pressure.**

| **Variable** | **Primary: any raised BP** | **Continuous BMI** | **Measured BP only** | **TRAP only** |
| --- | --- | --- | --- | --- |
| Age (per 10 years) | 1.19 (1.11–1.27)*** | 1.18 (1.11–1.26)*** | 1.18 (1.10–1.27)*** | 1.19 (1.10–1.29)*** |
| Male sex | 1.26 (1.08–1.47)** | 1.28 (1.10–1.49)** | 1.31 (1.09–1.57)** | 1.13 (0.97–1.33) |
| Obesity | 1.24 (1.09–1.42)** | — | 1.15 (0.98–1.36) | 1.26 (1.10–1.45)*** |
| Current smoking | 1.15 (0.91–1.45) | 1.12 (0.89–1.41) | 1.02 (0.76–1.38) | 1.19 (0.90–1.58) |
| Alcohol use | 1.11 (0.95–1.29) | 1.12 (0.97–1.31) | 1.16 (0.96–1.41) | 1.11 (0.95–1.30) |
| Site: Zvyagel | 1.05 (0.88–1.26) | 1.05 (0.88–1.26) | 0.93 (0.74–1.17) | 1.11 (0.92–1.34) |
| Site: Ternopil | 0.93 (0.78–1.12) | 0.93 (0.78–1.12) | 0.90 (0.73–1.11) | 0.93 (0.75–1.15) |
| Source: TCM | 0.81 (0.70–0.94)** | 0.81 (0.70–0.94)** | 0.99 (0.83–1.18) | — |
| Source: dental | 0.72 (0.55–0.94)* | 0.72 (0.55–0.95)* | 0.90 (0.68–1.20) | — |
| BMI (per 5 kg/m²) | — | 1.12 (1.05–1.19)*** | — | — |
| **No. of observations** | **341** | **341** | **340** | **147** |

Values are adjusted prevalence ratios (95% confidence intervals) from modified Poisson regression with robust variance. BMI, body-mass index; BP, blood pressure; TCM, traditional Chinese medicine; TRAP, cardiovascular-focused screening and referral stream. Column 1 is the primary model (composite outcome, all adults). Column 2 replaces the obesity indicator with continuous BMI. Column 3 uses the measured-only outcome (≥140/90 mmHg at the screening visit). Column 4 is restricted to the cardiovascular-focused stream. Reference categories are female sex, non-obese, never/former smoking, no alcohol use, Korosten (site), and the cardiovascular-focused stream (source). An em dash (—) indicates a term not included in that model. * P < 0.05; ** P < 0.01; *** P < 0.001.
