# Supplementary material for: Screen-detected raised blood pressure and gaps in the hypertension care cascade among adults attending a humanitarian medical mission in Ukraine: A cross-sectional study
Source: PLoS One. 2026 Sep 11;21(9):e0357957. doi: 10.1371/journal.pone.0357957 (PMC13567746; doi:10.1371/journal.pone.0357957)
Supplement: Supplementary file 3 [file pone.0357957.s003.docx]

**S1 Checklist. STROBE Statement — checklist of items that should be included in reports of cross-sectional studies.**

*Manuscript: “Screen-detected raised blood pressure and gaps in the hypertension care cascade among adults attending a humanitarian medical mission in Ukraine: a cross-sectional study.” Page numbers refer to the double-spaced manuscript file.*

| **Section/Topic** | **Item** | **Recommendation** | **Location in manuscript** |
| --- | --- | --- | --- |
| **Title and abstract** | 1 | (a) Indicate the study’s design with a commonly used term in the title or the abstract.  (b) Provide in the abstract an informative and balanced summary of what was done and what was found. | Title (p 1); Abstract (p 2) |
| **Introduction** | 2 | Background/rationale: Explain the scientific background and rationale for the investigation being reported. | Introduction (pp 3–4) |
|  | 3 | Objectives: State specific objectives, including any prespecified hypotheses. | Introduction, final paragraph (p 4) |
| **Methods** | 4 | Study design: Present key elements of study design early in the paper. | Methods — Study design and setting (p 4) |
|  | 5 | Setting: Describe the setting, locations, and relevant dates, including periods of recruitment, exposure, follow-up, and data collection. | Methods — Study design and setting (p 4) |
|  | 6 | Participants: (a) Give the eligibility criteria, and the sources and methods of selection of participants. | Methods — Participants (p 4) |
|  | 7 | Variables: Clearly define all outcomes, exposures, predictors, potential confounders, and effect modifiers. Give diagnostic criteria, if applicable. | Methods — Definitions and Measurements (pp 4–5) |
|  | 8* | Data sources/measurement: For each variable of interest, give sources of data and details of methods of assessment (measurement). Describe comparability of assessment methods if there is more than one group. | Methods — Measurements and Definitions (pp 4–5) |
|  | 9 | Bias: Describe any efforts to address potential sources of bias. | Methods — Definitions and Statistical analysis (p 5); Discussion — limitations (p 11) |
|  | 10 | Study size: Explain how the study size was arrived at. | Methods — Participants: all eligible attendees were included (self-selected convenience sample); no formal sample-size calculation was performed (p 4) |
|  | 11 | Quantitative variables: Explain how quantitative variables were handled in the analyses. If applicable, describe which groupings were chosen and why. | Methods — Statistical analysis: age per 10 years; obesity threshold and continuous BMI per 5 kg/m²; age bands (p 5) |
|  | 12 | Statistical methods: (a) Describe all statistical methods, including those used to control for confounding.  (b) Describe any methods used to examine subgroups and interactions.  (c) Explain how missing data were addressed.  (d) If applicable, describe analytical methods taking account of sampling strategy.  (e) Describe any sensitivity analyses. | Methods — Statistical analysis (p 5) |
| **Results** | 13* | (a) Report numbers of individuals at each stage of the study — e.g., numbers potentially eligible, examined for eligibility, confirmed eligible, included in the study, completing follow-up, and analysed.  (b) Give reasons for non-participation at each stage.  (c) Consider use of a flow diagram. | Methods — Participants and Results — Participants: 372 records, 8 excluded (age <18), 364 analysed; numbers reported in text (pp 4–5, 6). Non-participation not applicable (self-presentation); no flow diagram |
|  | 14* | (a) Give characteristics of study participants and information on exposures and potential confounders.  (b) Indicate number of participants with missing data for each variable of interest. | Results — Participants and Table 1 (p 6); missing outcome data (≈6%) noted in Methods — Statistical analysis (p 6) |
|  | 15* | Outcome data: Report numbers of outcome events or summary measures. | Results — Prevalence of raised blood pressure (p 7) and Table 1 (p 6) |
|  | 16 | (a) Give unadjusted estimates and, if applicable, confounder-adjusted estimates and their precision (e.g., 95% confidence intervals). Make clear which confounders were adjusted for and why they were included.  (b) Report category boundaries when continuous variables were categorized.  (c) If relevant, consider translating estimates of relative risk into absolute risk for a meaningful time period. | Results — Factors associated and Table 2 (p 8); category boundaries in Methods and Table 1 (pp 4–6); absolute treatment/control gaps in Care cascade (p 9) |
|  | 17 | Other analyses: Report other analyses done — e.g., analyses of subgroups and interactions, and sensitivity analyses. | Results — Sensitivity analyses and Care cascade (pp 9–10); S1 Table; S2 Table |
| **Discussion** | 18 | Key results: Summarise key results with reference to study objectives. | Discussion, first paragraph (p 10) |
|  | 19 | Limitations: Discuss limitations of the study, taking into account sources of potential bias or imprecision. Discuss both direction and magnitude of any potential bias. | Discussion — limitations paragraph (p 11) |
|  | 20 | Interpretation: Give a cautious overall interpretation of results considering objectives, limitations, multiplicity of analyses, results from similar studies, and other relevant evidence. | Discussion (pp 10–11) |
|  | 21 | Generalisability: Discuss the generalisability (external validity) of the study results. | Discussion — limitations and Conclusions (pp 11–12) |
| **Other information** | 22 | Funding: Give the source of funding and the role of the funders for the present study and, if applicable, for the original study on which the present article is based. | Financial Disclosure (submission system): MacKay Memorial Hospital grant 115012602; the funders had no role |

*Give information separately for exposed and unexposed groups, where applicable.

Note: An Explanation and Elaboration article discusses each checklist item and gives methodological background and published examples of transparent reporting. The STROBE checklist is best used in conjunction with this article (freely available on the Web sites of PLOS Medicine, Annals of Internal Medicine, and Epidemiology).
