# Supplementary material for: Screen-detected raised blood pressure and gaps in the hypertension care cascade among adults attending a humanitarian medical mission in Ukraine: A cross-sectional study
Source: PLoS One. 2026 Sep 11;21(9):e0357957. doi: 10.1371/journal.pone.0357957 (PMC13567746; doi:10.1371/journal.pone.0357957)
Supplement: Supplementary file 4 [file pone.0357957.s004.docx]

**S2 Checklist. PLOS questionnaire on inclusivity in global research.**

*Manuscript: “Screen-detected raised blood pressure and gaps in the hypertension care cascade among adults attending a humanitarian medical mission in Ukraine: a cross-sectional study” (PONE-D-26-30038). Page numbers refer to the double-spaced manuscript file.*

PLOS' policy on inclusivity in global research aims to improve transparency in the reporting of research performed outside of researchers' own country or community and ensures that PLOS publications reporting global research adhere to high standards for research ethics and authorship. The questions below outline ethical, cultural, and scientific considerations specific to inclusivity in global research. Questions that are not relevant to this study are marked “N/A”.

| **Question** | **Author response** |
| --- | --- |
| **Ethical considerations, permits and authorship (applicable to all research types)** | |
| Provide details as to who granted permissions and/or consent for the study to take place in the Methods section of your manuscript. This should include the names of all ethics boards, governmental organizations, community leaders or other bodies that provided approval for the study. If individuals provided approval refer to these people by their role or title but do not list their name(s). | **Reported on page number: 4**  The study was approved by the Institutional Review Board of MacKay Memorial Hospital, Taipei, Taiwan (approval number 25MMHIS075e; approved 19 May 2025). The mission itself was convened jointly with the Christian Medical Association of Ukraine, a Ukrainian national professional medical association, which arranged the three host sites – local church congregations at Korosten, Zvyagel and Ternopil – and whose leadership, together with the leadership of each host congregation, authorised the activity at each location.  Written informed consent was obtained from every participant before enrolment (Methods, Ethics, p 4). |
| If there were any deviations from the study protocol after approval was obtained please provide details of these changes in the Methods section of your manuscript. | **Reported on page number: N/A**  There were no deviations from the approved protocol. |
| Did this study involve local collaborators that are residents of the country where the research was conducted or members of the community studied? If you do not have any authors from said communities, please provide an explanation for this below.  *Everyone listed as an author should meet PLOS' criteria for authorship and all individuals who meet these criteria should be included in the author byline, rather than the acknowledgements. Authorship criteria is based on the International Committee of Medical Journal Editors (ICMJE) Uniform Requirements for Manuscripts Submitted to Biomedical Journals.* | Yes. The study was conducted jointly with Ukrainian collaborators, and a Ukraine-based collaborator is included in the author byline: Regina Maistrova (Christian Medical Association of Ukraine), who contributed to project administration and resources and is a resident of the country where the research was conducted.  Ukrainian clinical and administrative staff of the Christian Medical Association of Ukraine ran registration, interpretation and patient flow at all three sites (Korosten, Zvyagel, Ternopil). Those whose contributions did not meet ICMJE authorship criteria are recognised in the Acknowledgments. |
| **Human subjects research (e.g. health research, medical research, cross-cultural psychology)** | |
| Did you obtain written informed consent from a representative of the local community or region before the research took place? How did you establish who speaks for the community? Details of written informed consent obtained from study participants should be reported separately in the Methods section of your manuscript. | No written host agreement was signed with a community representative before the research took place. Permission to conduct the mission at each location was granted by the Christian Medical Association of Ukraine and by the leadership of the host church congregation at each of the three sites, but was not formalised in a signed document.  The reason is the operating environment. Security conditions in the affected regions changed substantially from day to day, and the choice of sites was in practice settled only about two to four weeks before the team's arrival, on security and logistical grounds. That planning window did not allow written host agreements to be negotiated in advance.  The Christian Medical Association of Ukraine was treated as the appropriate representative body because it is an established Ukrainian professional medical association with existing relationships with the host congregations and the communities in the regions served; it identified and arranged the host sites and convened the local clinical teams. At site level, the leadership of each host congregation gave permission for the mission to take place on its premises and for attendees to be invited.  Written informed consent was obtained from every individual participant before enrolment; this is reported separately in the Methods (Ethics, p 4). |
| How did members of the local community provide input on the aims of the research investigation, its methodology, and its anticipated outcome(s)? | The mission's clinical priorities, including the decision to add cardiovascular screening alongside the traditional Chinese medicine and dental streams, were set jointly with the Ukrainian partner organisation on the basis of needs its clinicians identified in the communities served.  Ukrainian collaborators reviewed the screening record form and the question set before the mission and advised on content, wording and length so that they were appropriate for the attending population, which included elderly residents and displaced people. The record form was produced in Ukrainian with English for internal reference.  A Ukraine-based co-author reviewed the study aims and the interpretation of the findings, and all authors approved the final manuscript. |
| When engaging with the local community, how did you ensure that the informed consent documents and other materials could be understood by local stakeholders? | All participant-facing materials, including the informed consent document and the study questionnaire, were provided in Ukrainian. Wording was drafted to be plain, courteous and suitable for elderly readers, and was checked by Ukrainian-speaking members of the partner organisation before use.  Ukrainian-speaking staff and interpreters were present at every station, so that participants could have the consent document and questions read aloud and explained, and could ask questions in Ukrainian before consenting. Consent was documented in writing. |
| Will the findings of the research be made available in an understandable format to stakeholders in the community where the study was conducted (e.g. via a presentation, summary report, copies of publications, etc.)? Please provide details of how this will be achieved. | Yes, and this has already begun. The aggregate statistical results were sent to the Christian Medical Association of Ukraine approximately one month after the team left Ukraine, that interval being the time required for data entry and statistical analysis. They were provided in English, the working language shared with the partner organisation's clinical staff, for circulation to the three host sites and the clinical teams involved.  A copy of the published open-access article will be shared with the Christian Medical Association of Ukraine and the host congregations once it appears.  At the individual level, findings were returned at the point of care: the mission's cardiovascular stream provided screening together with referral, so attendees were given their blood-pressure result during the visit and, where indicated, were directed to local services. The findings also inform the planning of subsequent missions to the same regions. |
| **Non-human subjects research using specimens/animals collected as part of the study, or those housed in archival collections (e.g. archaeology, paleontology, botany, zoology)** | |
| Did the permission you obtained from a local authority to perform the study include an agreement on access to outputs and benefit sharing? This may include procedures to enable fair distribution of the benefits and resources arising from the research performed. Please include any details of Prior Informed Consent and Benefit Sharing Agreements obtained. These may be required by field-specific regulations, for example the Convention on Biological Diversity (CBD) and the associated Nagoya Protocol. | N/A – this study involved human participants only. No specimens, animals, archival material or cultural artefacts were collected, imported, exported or analysed. |
| If the material used in your study was imported, please A) provide the year it was imported and B) indicate whether permits were obtained to import/export the materials used, C) provide details of any permits obtained. If this information is not available, please indicate this. | N/A – this study involved human participants only. No specimens, animals, archival material or cultural artefacts were collected, imported, exported or analysed. |
| If you used archival specimens, please state how the material used in your study was acquired by the institute it is held in and provide details of any permits obtained for the original excavations/sample collection. If this information is not available, please indicate this. | N/A – this study involved human participants only. No specimens, animals, archival material or cultural artefacts were collected, imported, exported or analysed. |
| How was the potential cultural significance of the materials collected in your study to local communities considered in your research design? Were Indigenous peoples and/or local researchers and institutions involved with archaeological excavations / collection of specimens? If so, please provide a description of their involvement. | N/A – this study involved human participants only. No specimens, animals, archival material or cultural artefacts were collected, imported, exported or analysed. |
| If your manuscript includes photographs of human remains please indicate whether authors obtained permission from descendants or affiliated cultural communities to do so. | N/A – the manuscript contains no photographs of human remains and no identifiable images of individuals. |

Completed by the corresponding author on behalf of all authors.
